# Translocation of *Mycobacterium tuberculosis* after experimental ingestion

**DOI:** 10.1371/journal.pone.0227005

**Published:** 2019-12-30

**Authors:** Mustapha Fellag, Ahmed Loukil, Jamal Saad, Hubert Lepidi, Fériel Bouzid, Fabienne Brégeon, Michel Drancourt

**Affiliations:** 1 IHU Méditerranée Infection, Marseille, France; 2 Aix-Marseille Univ., IRD, MEPHI, IHU Méditerranée Infection, Marseille, France; Rutgers Biomedical and Health Sciences, UNITED STATES

## Abstract

Human tuberculosis is a life-threatening infection following the inhalation of *Mycobacterium tuberculosis*, while the closely related bacteria *Mycobacterium bovis* and *Mycobacterium canettii* are thought to be transmitted by ingestion. To explore whether *M*. *tuberculosis* could also infect individuals by ingestion, male BALBc mice were fed 2 x 10^6^ CFUs of *M*. *tuberculosis* Beijing or phosphate-buffered saline as a negative control, over a 28-day experiment. While eight negative control mice remained disease-free, *M*. *tuberculosis* was identified in the lymph nodes and lungs of 8/14 mice and in the spleens of 4/14 mice by microscopy, PCR-based detection and culture. Whole-genome sequencing confirmed the identity of the inoculum and the tissue isolates. In these genetically identical mice, the dissemination of *M*. *tuberculosis* correlated with the results of the culture detection of four intestinal bacteria. These observations indicate that ingested *M*. *tuberculosis* mycobacteria can translocate, notably provoking lymphatic tuberculosis.

## Introduction

“The Lübeck disaster” was a dramatic episode of involuntary quasi-experimental deadly pulmonary tuberculosis following the ingestion of *Mycobacterium tuberculosis* [1]. It took place in Lübeck, Germany in 1929–1930, and a total of 251 neonates were given an oral vaccination with bacille Calmette-Guérin (BCG) that was inadvertently contaminated with various inocula of the virulent *M*. *tuberculosis* Kiel strain [1]. Subsequently, 228 (90%) neonates developed clinical tuberculosis, which presented as pharyngeal, abdominal and lymphatic tuberculosis in the vast majority of neonates; pulmonary tuberculosis developed in 13% of neonates, and 72 (28.7%) neonates died [1]. This carefully documented episode illustrated the potential for *M*. *tuberculosis* to cause deadly lymphatic and pulmonary tuberculosis after its ingestion under specific inoculation conditions in a pediatric population. Nevertheless, this episode did not further draw the attention of doctors, as it appeared to be an accident that did not reflect the natural history of pulmonary tuberculosis, which is considered to be an airborne-transmitted infection [2,3].

More recently, the emergence of primary lymph node tuberculosis due to *Mycobacterium canettii* in the Horn of Africa [4] and the re-emergence of primary pulmonary tuberculosis due to *Mycobacterium bovis* in Europe [5] has been observed. While the digestive route of contamination (foodborne disease) is suspected for *M*. *canettii* [4], ingestion is also the proven route of contamination for some cases of *M*. *bovis* tuberculosis in patients [6, 7].

These clinical observations directed our attention to question whether ingested *M*. *tuberculosis* could disseminate from the digestive tract to induce extra-digestive tuberculosis. Indeed, it was not possible to directly derive the behavior of *M*. *tuberculosis* from that of *M*. *canettii*, which is evolutionary distant from *M*. *tuberculosis* and encodes a ∼1.6% larger genome, offering a broader potential for transmission routes. As the question of translocation of *M*. *tuberculosis* was explored in very few and ancient experimental works in animals, results remain unclear [8–10]. We derived a mouse model of ingested *M*. *canettii* that we previously set up [11] to question whether ingested *M*. *tuberculosis* may induce systemic dissemination. Here we demonstrate the possible dissemination of *M*. *tuberculosis* after contamination by the oral route which raises the question of the role of *M*. *tuberculosis* translocation in the natural history of tuberculosis.

## Materials and methods

### Ethics statement

The experimental protocol, registered by the “Ministère de l’Enseignement Supérieur et de la Recherche” under reference no 235 2015092415474605, was approved by the Institutional Animal Care and Use Committee of Aix-Marseille University “C2EA-14”, France. The mice were handled according to the rules of Décret N° 2013–118, Février 7, 2013, France. All procedures on animals were performed in accordance with European law and agreed with Animal Research: Reporting In Vivo Experiments' (ARRIVE Guidelines http://www.nc3rs.org.uk). Mice were housed in individual plastic cages in a ventilated pressurized cabinet (A-BOX 160; Noroit, Rezé, France) with free access to sterile water and food. To limit the stress of animals, the environment was enriched with litter and cardboard tunnels. The behavior of the mice was observed daily for any signs of discomfort or distress (ruffled coat, hunched posture, lethargy). All efforts were made to limit the suffering of animals. The animals were sacrificed by cervical dislocation preceded by full general anesthesia (Sevoflurane). Then, the samples were collected post-mortem. All experiments were performed in a biosafety level 3 laboratory of the Institut Hospitalier Universitaire (IHU), Marseille, France.

### *M*. *tuberculosis* culture conditions and the preparation of bacterial inocula

*M*. *tuberculosis* Beijing strain was cultured in Middlebrook 7H10 (Becton Dickinson, Le Pont de Claix, France) supplemented with 10% oleic acid-albumin-dextrose catalase (OADC) (Becton Dickinson) for 30 days. The colonies were suspended in PBS, vigorously vortexed for 10 min using 3 mm sterile glass beads (Sigma-Aldrich, Saint-Quentin- Fallavier, France) and passed 10 times through a 25 G needle to disperse the clustered cells. The mycobacterial suspensions were then calibrated at 10^7^ CFU/mL using an optical density of 580 nm (Cell Density Meter; Fisher Scientific, Illkirch, France) and were confirmed by counting the mycobacteria after Ziehl-Neelsen staining.

### Mice infection protocol

A total of 22 eight-week-old BALB/CBYJ male mice (Charles River Laboratories, L'Arbresle, Lyon, France) weighing between 22 g and 24 g were housed in individual plastic cages (four to six animals per cage) placed in an isolator with free access to water and a standard diet. The mice were randomly allocated to the control group (n = 8) or to the *M*. *tuberculosis*-infected group (n = 14). Digestive inoculation was performed using 200 μL sterile PBS or mycobacterial suspension (equivalent to 2x10^6^ CFU) through sterile, single-use flexible feeding tubes for mouse oral gavage (Instech Laboratories, Inc., Plymouth Meeting, PA USA) as previously described [11], this method of gavage infection does not cause pain and does not require the use of anesthesia. To assess any possibility of procedure-induced lung contamination, two challenged mice were sacrificed just after inoculation, and their lungs and organs were sampled for PCR and culture analyses. The other mice were transferred to a safety cabinet with free access to food and water.

### Animal follow-up and tissue samplings

The animals were carefully monitored and weighed daily to assess their general condition and to look for endpoints. The endpoints were defined by the existence of at least one of the following signs: noisy breathing spontaneously audible by an experimenter standing near the cage, abdominal movements, head movements accompanying breathing, prostration and sluggishness, lack of feeding with a weight loss greater than or equal to 20% of their initial weight. A series of 4 infected mice were euthanized at 7 days, 14 days and 28 days p.i. A series of 2 control mice were euthanized at the same time points. The following organs were sampled: lungs, liver, spleen, lymph nodes, axillary lymph nodes, and kidneys. In the case of macroscopic abnormalities, an imprint slide of the organ was directly performed. Fresh organs were cut, and the inside surface of the tissue section was dabbed against a clean glass slide. Fresh stool was sampled directly from the mouse distal colon after laparotomy and intestine dissection. The samples were stored at –20°C for further analyses.

### Pathological examination and FISH

For each sacrificed mouse, the liver, lungs, kidneys, heart, and lymph nodes were fixed with 4% buffered formalin and were embedded in paraffin. Sections (3 μm) of these specimens were obtained for routine hematoxylin-eosin-saffron staining. Granulomas were defined as collections of ten or more macrophages and lymphocytes within the organs. The specific detection of *M*. *tuberculosis* in the lung and lymph node tissues was performed on imprint slides combining FISH and Ziehl-Neelsen staining as previously described with some modifications [12]. Briefly, the imprint slides were heat-fixed at 90°C for 30 min and were flooded with 4% formaldehyde (Sigma-Aldrich, Saint-Quentin-Fancy, France) for 30 min; then, the slides were submitted for FISH and Ziehl-Neelsen staining. Slides were incubated with 10 mg/mL of lysozyme solution for 30 minutes at 37°C with (Sigma-Aldrich, Saint-Quentin-Fancy, France) and 5 μg/mL of proteinase K (Sigma-Aldrich) for 5 min at 37°C. After washing with distilled water, slides were covered with a hybridization solution containing the red/orange fluorescent oligonucleotide probe (5'- Alexa-555-AGCGGGGTGATGTCAACCCAG-3') targeting *M*. *tuberculosis* complex *rpoB* gene (10 μmol/L) and incubated at 65°C for 10 minutes then at 37°C overnight. Finally, slides were serially washed with saline-sodium citrate (SSC) buffer, stained with a cold Ziehl-Neelsen staining (Kit Quick-TB, RAL DIAGNOSTICS, Martillac, France) and mounted with ProLong Diamond Antifade (Fisher Scientific) containing 4',6-diamidino-2-phenylindole (DAPI). Microscopic observation was performed using a 100X oil immersion objective of a Leica DMI6000 fluorescence microscope.

### PCR-based experiments

Molecular PCR-based assays were used to confirm the identification of cultured colonies and to quantify the *M*. *tuberculosis* load into crushed organs as previously described with modifications [11]. Briefly, for positive cultures, the colonies were inactivated in 150 μL PBS by incubation at 56°C for three hours with 150 μL G2 lysis buffer and 15 μL proteinase K (20 mg/mL); then, the colonies were broken with glass powder using a FastPrep instrument (MP Biomedical Europe, Illkirch, France) at a speed of 6.5 m/s for 90 s. The DNA was extracted using an EZ1 DNA Tissue Kit (Qiagen, Hilden, Germany). Quantitative real-time PCR (qPCR) was performed using the CFX96 thermocycler (Bio-Rad, Hercules, CA, USA). The qPCR reagents, primers and probes were incorporated as previously described [13]. The quantification of *M*. *tuberculosis* in culture-positive tissues was performed with molecular detection by measuring the DNA copy number of mycobacteria divided by the number of described [11]. The tissue DNA was extracted as follows: aliquots of 150 μL were incubated overnight at 56°C with 150 μL of G2 buffer mixed with 15 μL proteinase K (20 mg/mL). After two cycles of mechanical lysis (45 s), the total DNA was extracted using the EZ1 DNA Tissue Kit. DNA extraction from stools was performed using a QIAamp® DNA Stool Kit (Qiagen). Two qPCR assays were performed targeting the *M*. *tuberculosis* internal transcribed spacer (ITS) [13] and the housekeeping mouse hydroxyl-methylbilane synthase gene (*HMBS)* for normalization as previously described [14]. A standard curve of *M*. *tuberculosis* ITS qPCR was generated using the extracted DNA from 3-fold serial dilutions of *M*. *tuberculosis* Beijing suspensions (10^7^ CFUs/mL to 1 CFU/mL).

### Bacterial culture

Frozen organs were thawed, lymph node material was suspended in 250 μL of sterile PBS, and the other organs were suspended in 500 μL and were mechanically grounded. The cultures were inoculated and were incubated in a Middlebrook 7H10—OADC (Becton Dickinson) for two months at 37°C. When the colonies were visualized, the gelose surface was carefully sampled, and the material was processed for matrix-assisted laser desorption/ionization mass spectrometry (MALDI-TOF-MS) (Microflex, Brucker Daltonics®) and real-time PCR analysis.

### Genome sequencing

The total DNA was extracted using an InstaGen matrix (Bio-Rad, Marnes-la-Coquette, France) from the *M*. *tuberculosis* inoculum and from one isolate made from one cervical lymph node, one mesenteric lymph node and one lung. A total of 200 μL of IntsaGen matrix was added to the pellet of each isolation and was incubated at 56°C for 30 min; then, glass beads were added. We vortexed at a high speed for 10 seconds, and we placed the tube in a 100°C heat block for 10 min. Next, a mechanical treatment by a FastPrep BIO 101 instrument (Qbiogene Strasbourg, France) was performed at maximum speed (6.5 m/sec) for 45 seconds to stain the membrane. Finally, the tube was centrifuged for 15 min, and the DNA in the supernatant was ready for sequencing. The DNA extracted was quantified by a Qubit assay with a High Sensitivity Kit (Life Technologies, Carlsbad, CA, USA), and 0.2 μg/μL of DNA was sequenced by an Illumina MiSeq (Illumina Inc., San Diego, USA). The DNA was fragmented and amplified by limited PCR (12 cycles) to introduce dual-index barcodes and sequencing adapters. After purification with AMPure XP beads (Beckman Coulter Inc., Fullerton, CA, USA), the libraries were normalized and pooled for sequencing on a MiSeq instrument. Paired-end sequencing and automated cluster generation with dual indexed 2 × 251 bp reads. The total data obtained was 10.3 Gb from a 564 k/mm^2^ cluster density with cluster passing quality control filters of 96.1%. The genomic reads of each isolate were assembled using SPAdes software [15] and were annotated using [16]. The Total Genotyping Solution for *M*. *tuberculosis* online tool was used for the identification of lineages and sublineages using the KvarQ script [17]. Using the same tool, *in silico* IS*6110* insertion and spoligotyping were detected. The identification results were further supported by TB Profiler and Mykrobe Predictor-TB [18, 19]. The genome sequences have been deposited into EBI under the following accession numbers: PRJEB31649-1 (*Mycobacterium tuberculosis* inoculum), PRJEB31649-2 (*Mycobacterium tuberculosis* cervical lymph node isolate), LR536056.1 (*Mycobacterium tuberculosis* mesenteric lymph node isolate) and PRJEB31649-4 (*Mycobacterium tuberculosis* lung isolate).

### Statistics

The data were input into Microsoft Excel for Office 365 to estimate the means and standard deviations. A chi-square statistical test was performed using RStudio-R software (https://www.rstudio.com), and a P value < 0.05 was considered significant.

## Results

### Ingested *M*. *tuberculosis* infects the digestive tract in mice

**Fig 1:**
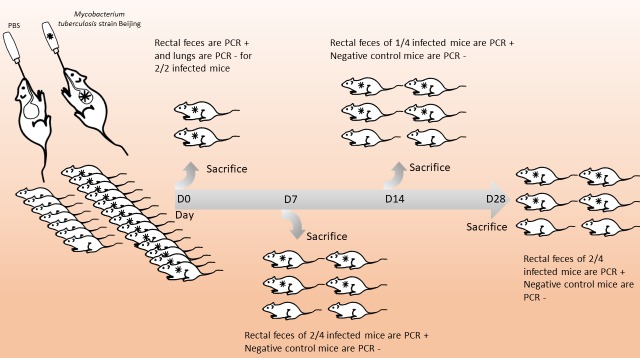
Infection of the digestive tract in a mouse model of ingested *M*. *tuberculosis*.

A total of 14 male BALBc mice were inoculated with the *M*. *tuberculosis* Beijing strain by the gastric route after the inoculum’s resistance to acidity and biliary salts was verified in the presence of 8 negative control mice given sterile phosphate-buffered saline (PBS). After two mice were sacrificed four hours post-challenge to verify the absence of inhaled *M*. *tuberculosis*, the 12 remaining mice were apparently healthy and showed no sign of pain and no weight loss was observed over the 28-day experiment. In these 12 mice, *M*. *tuberculosis* was detected by PCR in the rectal feces collected from the sacrificed mice at day 7 (2/4 mice), day 14 (1/4) and day 28 (2/4), confirming that ingested *M*. *tuberculosis* was transmitted and persisted along the entire digestive tract of the challenged mice (Fig 1).

### Ingested *M*. *tuberculosis* infects the lymph nodes and lungs of mice

**Fig 2:**
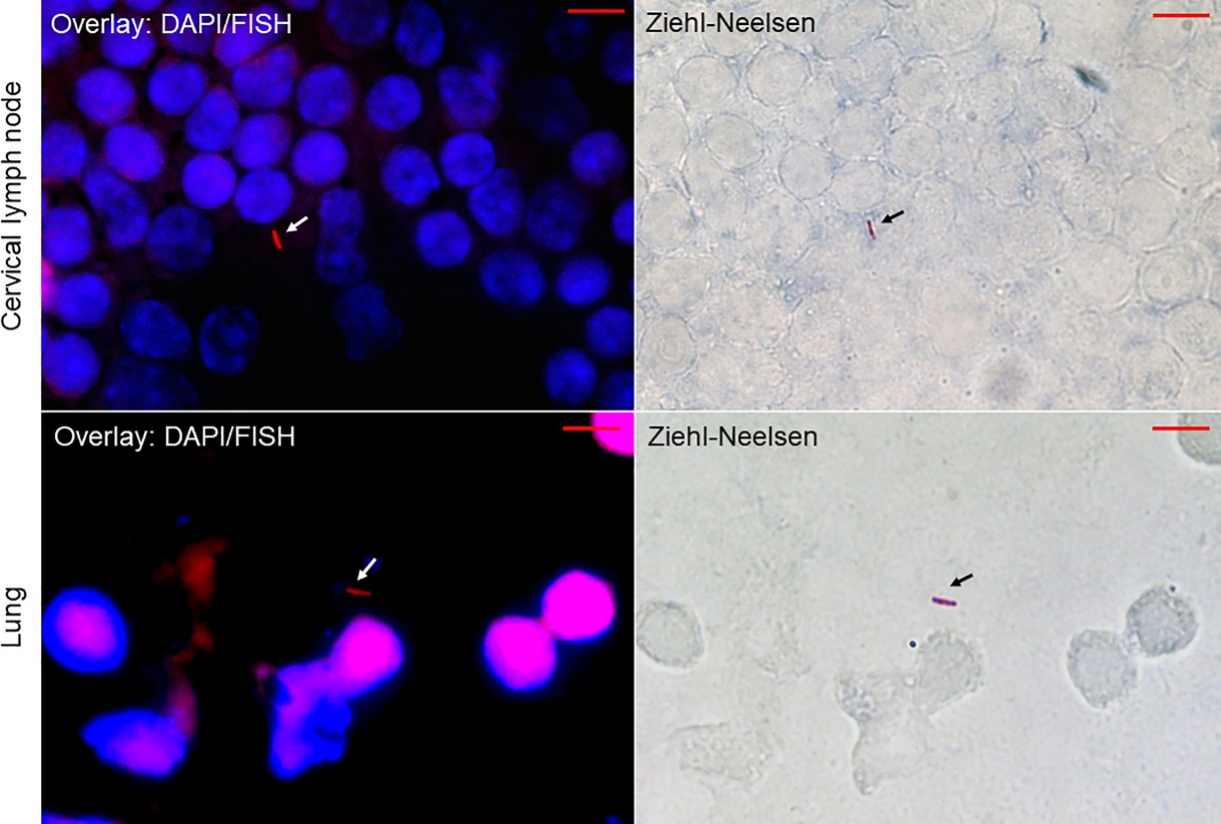
Microscopic images of imprint slides prepared from lung and cervical lymph nodes of *Mycobacterium tuberculosis*-infected mice at day 28 post-infection with combined FISH, DAPI and Ziehl-Neelsen staining. For fluorescent *in situ* hybridization (FISH), the slides were observed using a red channel of a Leica DMI6000 microscope under a 100 X oil-immersion objective. The images were captured in the same microscopic field using a Hamamatsu Orca AG camera (Hamamatsu Photonics, Herrsching-am-Ammersee, Germany) for FISH-positive mycobacteria (left images, white arrows) and a DFC425 C Digital Microscope Camera (Leica Microsystemes, Nanterre, France) for Ziehl-Neelsen-positive mycobacteria (right images, black arrows). Scale bar = 5 μm.

Enlarged cervical, tracheobronchial and mediastinal lymph nodes were observed in 3/4 mice sacrificed at day 7, in 2/4 mice sacrificed at day 14 and in 2/4 mice sacrificed at day 28. Macroscopic changes were observed with variable degrees of congestion in the lungs of all infected mice, while the livers, spleens, kidneys and digestive tracts had a normal appearance in all mice. Accordingly, pathological examination found no detectable granulomas, inflammatory infiltrates or necrotic damages in any organ. These observations agree with previous observations in the experimental models of pulmonary tuberculosis after tracheal instillation and aerosols [20, 21]. However, microscopic observations confirmed the dissemination of *M*. *tuberculosis* in the lung and lymph node tissues on post-infection day 28, with the mycobacteria appearing as Ziehl-Neelsen-positive rods that were fluorescing in red due to the use of fluorescent *in situ* hybridization (FISH) staining [12] (Fig 2, S1 Fig). These mycobacteria were confirmed to be *M*. *tuberculosis* and were quantified by quantitative real-time PCR (qPCR) (Table 1, S1 Table). The spleen and cervical lymph nodes exhibited the highest load of mycobacteria after 14 days and 28 days of infection. The mycobacterial load was 5 ± 0.9 mycobacteria/10 spleen cells and 3 ± 9 mycobacteria/10 lymph node cells at day 14 post-infection and was 4 ± 26 mycobacteria/10 spleen cells and 0.6 ± 0.3 mycobacteria/10 lymph node cells at day 28 post-infection. In the lungs, the highest *M*. *tuberculosis* burden was measured at day 14 post-infection, and the lowest burden was measured at day 7 post-infection; the mycobacterial loads were 11 ± 13 and 1.2 ± 0.7 mycobacteria/10^6^ cells, respectively. All the samples collected immediately after gavage in the inoculated and control animals remained negative according to the culture and PCR analyses, except for the digestive tract samples. Finally, the viability of detected *M*. *tuberculosis* mycobacteria was assessed by culture (Table 1) as follows: on day 7, *M*. *tuberculosis* was cultured from the lungs, the cervical and mesenteric lymph nodes and the stool in two out of four mice. Additionally, one mouse showed an *M*. *tuberculosis*-positive axillary lymph node. At day 14, *M*. *tuberculosis* was cultured from 3 of the 4 sacrificed mice; from the lungs, stools, spleens, cervical and mesenteric lymph nodes and livers of two of these three mice; and from the mediastinal and tracheobronchial lymph nodes of one of these three mice. In the last group of 4 mice sacrificed at 28 days post-infection, *M*. *tuberculosis* was cultured from the lungs, spleen and cervical lymph nodes of three mice and in the oesotracheal lymph nodes and livers of two mice. In one mouse, the culture was *M*. *tuberculosis*-positive in the mediastinal and mesenteric lymph nodes, in the liver and in the stool. *M*. *tuberculosis* was not cultured in any of the eight negative control mice (Table 1). Comparing the whole genome sequence of the inoculum with that derived from the lymph node and lung isolates indicated the same 2.2.1 sublineage in these four isolates. Core-genome analysis showed that the four strains were grouped into a unique cluster with the reference *M*. *tuberculosis* lineage 2.2.1, sharing the same position as IS*6110* and the same spoligotyping pattern. These observations confirmed the translocation of viable *M*. *tuberculosis* beyond the lymphatic system to several organs, including the lungs.

**Table 1:** Culture result and microbial load data^a^ for organs sampled from mice challenged with *M*. *tuberculosis*.

Mouse number	Days post- infection	Lung		Cervical lymph nodes		Axillary lymph nodes		Oesotracheal/mediastinal lymph nodes		Mesenteric lymph nodes		Liver		Spleen	
		Culture	qPCR	Culture	qPCR	Culture	qPCR	qPCR	Culture	Culture	qPCR	Culture	qPCR	Culture	qPCR
1	0	-	-	-	-	-	-	-	-	-	-	-	-	-	-
2	0	-	-	-	-	-	-	-	-	-	-	-	-	-	-
3	7	-	-	+	1.59E-04	+	4.55E-05	-	-	-	-	-	-	-	-
4	7	-	-	-	-	-	-	-	-	-	-	-	-	-	-
5	7	+	1.84E-06	-	-	-	-	-	-	+	6.67E-07	-	-	-	-
6	7	+	7.47E-07	+	8.13E-06	-	1.23E-03	-	-	+	7.97E-07	-	-	-	-
**7**	14	-	2.80E-05	+	1.54E+00	-	-	-	-	+	1.03E-01	+	5.24E-06	-	-
**8**	14	-	-	-	-	-	-	-	-	-	-	-	-	-	-
**9**	14	+	9.39E-06	+	2.05E-01	-	-	-	-	+	2.95E-05	-	-	+	5.90E-01
**10**	14	+	1.57E-06		-	-	-	4.10E-05	+	-	-	+	1.20E-03	+	4.64E-01
11	28	+	3.45E-06	+	1.88E-02	-	-	-	-	-	-	-	-	+	3.25E-02
12	28	+	7.92E-05	+	2.32E-02	-	-	4.79E-06	+	-	-	-	-	+	2.32E-01
13	28	-	-	-	-	-	-	-	-	-	-	-	-	-	-
14	28	+	1.32E-06	+	7.47E-02	-	-	1.92E-04	+	+	8.83E-06	+	8.62E-07	+	4.72E+00

^a^qPCR data are the number of mycobacteria/mouse cell.

## Discussion

Ingested *M*. *tuberculosis* disseminated to lymph nodes and the lungs in the experimental laboratory animals as was verified by the *M*. *tuberculosis*-negative state of the negative controls, the concordant results obtained by the independent methods of observation and the reproducibility of the results. The fact that *M*. *tuberculosis* mycobacteria was not retrieved in the lungs in animals sacrificed within four hours post-challenge, demonstrate that the observations here reported do not result from the mere inhalation of the mycobacteria. Pulmonary infection observed in this experimental model was characterized by congestive lungs containing viable and culturable *M*. *tuberculosis*. These observations matched those issued from previously reported experimental mouse models. In particular, we found no significant difference (P = 0.336) in terms of overall four-week post-infection survival and *M*. *tuberculosis* dissemination in the lungs of the challenged mice [21–24]. It has been reported that no granulomas were observable before 30 days post-infection in *M*. *tuberculosis*-infected mice; this is in line with our observations [21]. This indicates that the model reported here is valuable and produces a gross pathology indistinguishable from that previously reported in male mice that were experimentally infected with *M*. *tuberculosis*. However, mice challenged by the digestive route developed more diseased lymph nodes earlier in the infection than those in previously reported aerosol models [25]. These results suggest that *M*. *tuberculosis* translocated from the digestive tract to the lungs through the lymphatic system, then followed by a progressive clearance of *M*. *tuberculosis* by the mouse immune system. Altogether, our observations are in agreement with the previously reported observations that indicate that the route of *M*. *tuberculosis* inoculation may govern the clinical form of tuberculosis.

Here, we challenged genetically homogeneous mice to bypass the genetic control of the infection [26]. We observed that only half of the mice had a lung dissemination of *M*. *tuberculosis* despite all challenged mice having the same genetic background (BALBc male mice). Considering that digestive tract microbiota is part of the individual [27], we suspected that subtle differences in the digestive tract microbiota could correlate with the dissemination of *M*. *tuberculosis* by the lymphatic route. Indeed, the mouse stool microbiota was shown to be altered after *M*. *tuberculosis* aerosol experimental infection, by metagenomics analyses [28]. Also, altered gut microbiota was observed in patients infected with *M*. *tuberculosis* [29] along with the excretion of viable mycobacteria in the stool [30]. In some populations, the epidemiology of tuberculosis is currently shifting from pulmonary to lymph node tuberculosis. As an example in Tunisia, increasing the extra-pulmonary/pulmonary tuberculosis ratio from 19.6% in 1996 to 32.6% in 2007 was driven by lymph node tuberculosis, the second most common form that causes 23% of cases [26]. Likewise, infections of *M*. *bovis* and *M*. *canettii* from the digestive route promote a predominantly lymphatic form of infection [11]. These observations and the ones reported here suggest that the current epidemiological shift of tuberculosis reported in some countries may be partially driven by the changing modes of contamination in populations, from the classic airborne transmission to digestive transmission. This hypothesis warrants further field studies including that of the digestive tract microbiota by culturomics [31].

## Conclusion

The observations reported here enrich the literature to indicate that ingested *M*. *tuberculosis* promotes lymphatic and pulmonary tuberculosis. The experimental observations reported here fully agree with the clinical observations made during the “Lübeck disaster”, where almost all the neonates contaminated by the oral route with *M*. *tuberculosis* developed lymphadenopathies; this was also the most salient clinical feature in this episode [1].

These observations may change our common view of the natural history of tuberculosis as an exclusively air-borne infection. In particular, the experimental results here reported question the potential of recirculation of viable *M*. *tuberculosis*, otherwise routinely detected in the stools of pulmonary tuberculosis patients [30].

Translocation is therefore a common trait of the *M*. *tuberculosis* complex, previously observed for *M*. *bovis* [6, 7] and for *M*. *canettii* [11]. These observations suggest that translocation was shared by the common ancestor of the *M*. *tuberculosis* complex and was conserved along with the co-evolution of tuberculosis and *Homo sapiens* [32, 33]. In the late Pleistocene, hunter-gatherer populations of *H*. *sapiens* were very thinly spread [34] and unlikely to reach the critical mass necessary to allow tuberculosis evolution from an environmental ancestor. Pleistocene hunter-gatherers could possibly have contracted tuberculosis from environmental sources such as infected meat and other animal products in a first step, then following an oral-fecal route during the first times of sedentarization. Indeed, *M*. *tuberculosis* is routinely cultured in the stools of patients with pulmonary tuberculosis, confirming the viability of *M*. *tuberculosis* in the digestive tract of these patients [30]; viability known to be preserved for months in *M*. *tuberculosis*-contaminated soil [35].

Whether oral route contamination still translates in the actual epidemiology of tuberculosis in some populations, in particular in populations known to be dually exposed to tuberculosis and enteric pathogens with a human reservoir such as typhoid fever, warrants further investigations. As part of such investigations, culturomic investigations of the digestive tract microbiota may provide clues to the epidemiological shift from pulmonary to lymphatic tuberculosis, which is currently being observed in some populations.

## Supporting information

S1 Fig Microscopic images of imprint slides prepared from lung and cervical lymph nodes of *Mycobacterium tuberculosis*-infected mice at day 28 post-infection with combined FISH, DAPI and Ziehl-Neelsen staining. The slides were observed using a Leica DMI6000 microscope under a 100 X oil-immersion objective. The images were captured in the same microscopic field for FISH-positive mycobacteria (white arrows) and Ziehl-Neelsen-positive mycobacteria (black arrows). Scale bar = 5 μm.
(TIF)

S1 Table Quantification data of *M. tuberculosis* load per organ cells. (XLSX)

## References

[1] Fox GJ, Orlova M, Schurr E. Tuberculosis in Newborns: The Lessons of the “Lübeck Disaster” (1929–1933). PLOS Pathogens. 2016;12: e1005271 10.1371/journal.ppat.1005271 26794678 PMC4721647

[2] Escombe AR, Oeser C, Gilman RH, Navincopa M, Ticona E, Martínez C, et al The Detection of Airborne Transmission of Tuberculosis from HIV-Infected Patients, Using an In Vivo Air Sampling Model. Clin Infect Dis. 2007;44: 1349–1357. 10.1086/515397 17443474 PMC2912511

[3] Turner RD, Bothamley GH. Cough and the Transmission of Tuberculosis. J Infect Dis. 2015;211: 1367–1372. 10.1093/infdis/jiu625 25387581

[4] Aboubaker Osman D, Bouzid F, Canaan S, Drancourt M. Smooth Tubercle Bacilli: Neglected Opportunistic Tropical Pathogens. Front Public Health. 2016;3 10.3389/fpubh.2015.00283 26793699 PMC4707939

[5] Evans JT, Smith EG, Banerjee A, Smith RM, Dale J, Innes JA, et al Cluster of human tuberculosis caused by *Mycobacterium bovis*: evidence for person-to-person transmission in the UK. The Lancet. 2007;369: 1270–1276. 10.1016/S0140-6736(07)60598-4 17434402

[6] Smith RMM, Drobniewski F, Gibson A, Montague JDE, Logan MN, Hunt D, et al *Mycobacterium bovis* Infection, United Kingdom. Emerg Infect Dis. 2004;10: 539–541. 10.3201/eid1003.020819 15109433 PMC3322792

[7] Grange JM. *Mycobacterium bovis* infection in human beings. Tuberculosis. 2001;81: 71–77. 10.1054/tube.2000.0263 11463226

[8] Calmette, M.A. The intestinal origin of pulmonary tuberculosis, and the mechanism of tuberculous infection. Reviews. Agriculture and Fisheries Annual Reports of Proceedings under the Diseases of Animal Actes, for the year 1905. 1905.

[9] Pierce C, Dubos RJ, Middlebrook G. Infection of Mice with Mammalian Tubercle Bacilli Grown in Tween-Albumin Liquid Medium. Journal of Experimental Medicine. 1947;86: 159–174. 10.1084/jem.86.2.159 19871664 PMC2135714

[10] Lefford M.J. Diseases in mice and rats In Kubica GP and Wayne LG (ed), The mycobacteria, New York Pp. 947–977. 1984.

[11] Bouzid F, Brégeon F, Lepidi H, Donoghue HD, Minnikin DE, Drancourt M. Ready Experimental Translocation of *Mycobacterium canettii* Yields Pulmonary Tuberculosis. Infect Immun. 2017;85 10.1128/IAI.00507-17 28923895 PMC5695119

[12] Loukil A, Kirtania P, Bedotto M, Drancourt M. FISHing *Mycobacterium tuberculosis* Complex by Use of a rpoB DNA Probe Bait. Journal of Clinical Microbiology. 2018;56: e00568–18. 10.1128/JCM.00568-18 30068538 PMC6156301

[13] Bruijnesteijn Van Coppenraet ES, Lindeboom JA, Prins JM, Peeters MF, Claas ECJ, Kuijper EJ. Real-time PCR assay using fine-needle aspirates and tissue biopsy specimens for rapid diagnosis of mycobacterial lymphadenitis in children. J Clin Microbiol. 2004;42: 2644–2650. 10.1128/JCM.42.6.2644-2650.2004 15184446 PMC427856

[14] Ding S, Chi MM, Scull BP, Rigby R, Schwerbrock NMJ, Magness S, et al High-fat diet: bacteria interactions promote intestinal inflammation which precedes and correlates with obesity and insulin resistance in mouse. PLoS ONE. 2010;5: e12191 10.1371/journal.pone.0012191 20808947 PMC2922379

[15] Bankevich A, Nurk S, Antipov D, Gurevich AA, Dvorkin M, Kulikov AS, et al SPAdes: A New Genome Assembly Algorithm and Its Applications to Single-Cell Sequencing. Journal of Computational Biology. 2012;19: 455–477. 10.1089/cmb.2012.0021 22506599 PMC3342519

[16] Seemann T. Prokka: rapid prokaryotic genome annotation. Bioinformatics. 2014;30: 2068–2069. 10.1093/bioinformatics/btu153 24642063

[17] Steiner A, Stucki D, Coscolla M, Borrell S, Gagneux S. KvarQ: targeted and direct variant calling from fastq reads of bacterial genomes. BMC Genomics. 2014;15: 881 10.1186/1471-2164-15-881 25297886 PMC4197298

[18] Coll F, McNerney R, Preston MD, Guerra-Assunção JA, Warry A, Hill-Cawthorne G, et al Rapid determination of anti-tuberculosis drug resistance from whole-genome sequences. Genome Med. 2015;7: 51 10.1186/s13073-015-0164-0 26019726 PMC4446134

[19] Coll F, McNerney R, Guerra-Assunção JA, Glynn JR, Perdigão J, Viveiros M, et al A robust SNP barcode for typing *Mycobacterium tuberculosis* complex strains. Nature Communications. 2014;5: 4812 10.1038/ncomms5812 25176035 PMC4166679

[20] Dormans J, Burger M, Aguilar D, Hernandez-Pando R, Kremer K, Roholl P, et al Correlation of virulence, lung pathology, bacterial load and delayed type hypersensitivity responses after infection with different *Mycobacterium tuberculosis* genotypes in a BALB/c mouse model. Clin Exp Immunol. 2004;137: 460–468. 10.1111/j.1365-2249.2004.02551.x 15320894 PMC1809137

[21] Jeon B-Y, Kwak J, Hahn M-Y, Eum S-Y, Yang J, Kim S-C, et al In vivo characteristics of Korean Beijing *Mycobacterium tuberculosis* strain K1 in an aerosol challenge model and in the Cornell latent tuberculosis model. Journal of Medical Microbiology. 2012;61: 1373–1379. 10.1099/jmm.0.047027-0 22820694

[22] Dibbern J, Eggers L, Schneider BE. Sex differences in the C57BL/6 model of *Mycobacterium tuberculosis* infection. Sci Rep. 2017;7: 10957 10.1038/s41598-017-11438-z 28887521 PMC5591305

[23] Medina E, North RJ. Resistance ranking of some common inbred mouse strains to *Mycobacterium tuberculosis* and relationship to major histocompatibility complex haplotype and Nramp1 genotype. Immunology. 1998;93: 270–274. 10.1046/j.1365-2567.1998.00419.x 9616378 PMC1364188

[24] Dunn PL, North RJ. Virulence ranking of some *Mycobacterium tuberculosis* and *Mycobacterium bovis* strains according to their ability to multiply in the lungs, induce lung pathology, and cause mortality in mice. Infect Immun. 1995;63: 3428–3437. 7642273 10.1128/iai.63.9.3428-3437.1995 PMC173472

[25] Chackerian AA, Alt JM, Perera TV, Dascher CC, Behar SM. Dissemination of *Mycobacterium tuberculosis* is influenced by host factors and precedes the initiation of T-cell immunity. Infect Immun. 2002;70: 4501–4509. 10.1128/IAI.70.8.4501-4509.2002 12117962 PMC128141

[26] Smaoui S, Mezghanni MA, Hammami B, Zalila N, Marouane C, Kammoun S, et al Tuberculosis lymphadenitis in a southeastern region in Tunisia: Epidemiology, clinical features, diagnosis and treatment. International Journal of Mycobacteriology. 2015;4: 196–201. 10.1016/j.ijmyco.2015.04.004 27649866

[27] Lagier J-C, Dubourg G, Million M, Cadoret F, Bilen M, Fenollar F, et al Culturing the human microbiota and culturomics. Nat Rev Microbiol. 2018; 540–550. 10.1038/s41579-018-0041-0 29937540

[28] Winglee K, Eloe-Fadrosh E, Gupta S, Guo H, Fraser C, Bishai W. Aerosol *Mycobacterium tuberculosis* Infection Causes Rapid Loss of Diversity in Gut Microbiota. PLOS ONE. 2014;9: e97048 10.1371/journal.pone.0097048 24819223 PMC4018338

[29] Luo M, Liu Y, Wu P, Luo D-X, Sun Q, Zheng H, et al Alternation of Gut Microbiota in Patients with Pulmonary Tuberculosis. Front Physiol. 2017;8: 822 10.3389/fphys.2017.00822 29204120 PMC5698276

[30] El Khéchine A, Henry M, Raoult D, Drancourt M. Detection of *Mycobacterium tuberculosis* complex organisms in the stools of patients with pulmonary tuberculosis. Microbiology (Reading, Engl). 2009;155: 2384–2389. 10.1099/mic.0.026484-0 19389783

[31] Lagier J-C, Hugon P, Khelaifia S, Fournier P-E, Scola BL, Raoult D. The Rebirth of Culture in Microbiology through the Example of Culturomics To Study Human Gut Microbiota. Clinical Microbiology Reviews. 2015;28: 237–264. 10.1128/CMR.00014-14 25567229 PMC4284300

[32] Comas I, Coscolla M, Luo T, Borrell S, Holt KE, Kato-Maeda M, et al Out-of-Africa migration and Neolithic coexpansion of *Mycobacterium tuberculosis* with modern humans. Nat Genet. 2013;45: 1176–1182. 10.1038/ng.2744 23995134 PMC3800747

[33] Comas I, Hailu E, Kiros T, Bekele S, Mekonnen W, Gumi B, et al Population Genomics of *Mycobacterium tuberculosis* in Ethiopia Contradicts the Virgin Soil Hypothesis for Human Tuberculosis in Sub-Saharan Africa. Curr Biol. 2015;25: 3260–3266. 10.1016/j.cub.2015.10.061 26687624 PMC4691238

[34] Stewart JR, Stringer CB. Human evolution out of Africa: the role of refugia and climate change. Science. 2012;335: 1317–1321. 10.1126/science.1215627 22422974

[35] Ghodbane R, Mba Medie F, Lepidi H, Nappez C, Drancourt M. Long-term survival of tuberculosis complex mycobacteria in soil. Microbiology. 2014;160: 496–501. 10.1099/mic.0.073379-0 24425768

