# Supplementary figures and images for: Translocation of Mycobacterium tuberculosis after experimental ingestion
Source: PLoS One. 2019 Dec 30;14(12):e0227005. doi: 10.1371/journal.pone.0227005 (PMC6936814; doi:10.1371/journal.pone.0227005)

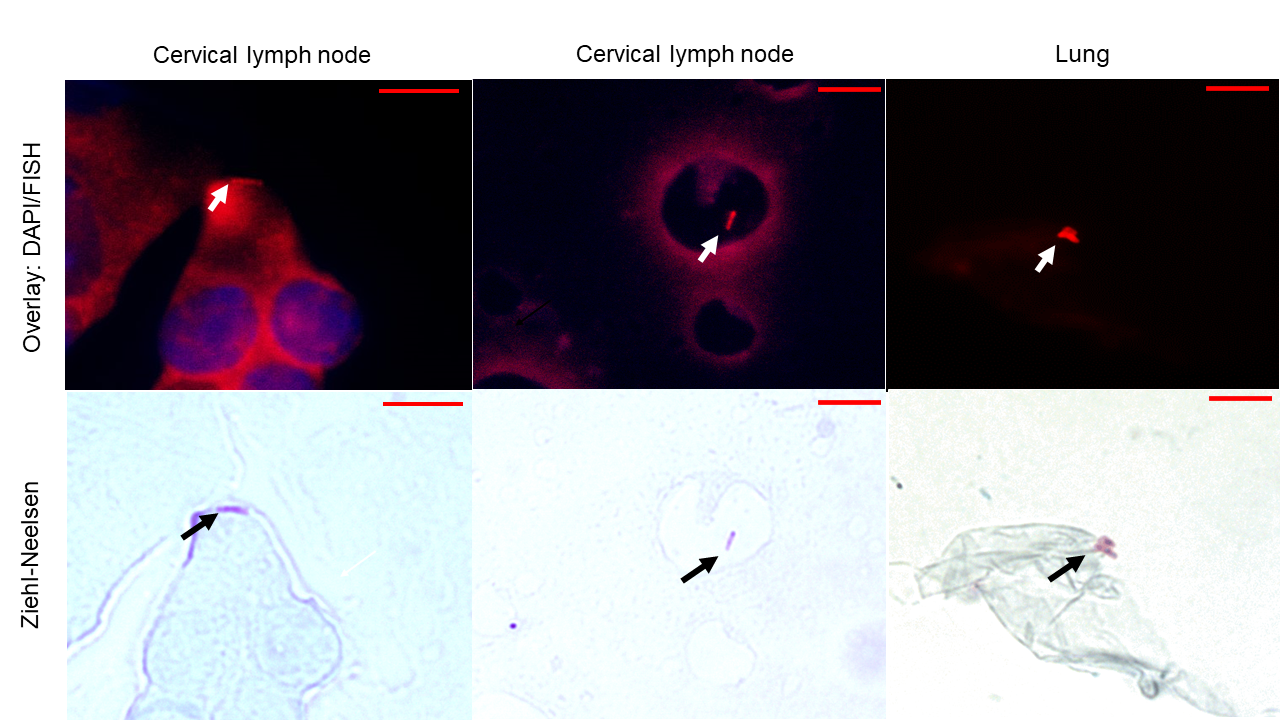

Supplement: S1 Fig — The slides were observed using a Leica DMI6000 microscope under a 100 X oil-immersion objective. The images were captured in the same microscopic field for FISH-positive mycobacteria (white arrows) and Ziehl-Neelsen-positive mycobacteria (black arrows). Scale bar = 5 μm. (TIF) [file pone.0227005.s001.tif]
